# ‘Turning the tide’ on hyperglycemia in pregnancy: insights from multiscale dynamic simulation modeling

**DOI:** 10.1136/bmjdrc-2019-000975

**Published:** 2020-05-31

**Authors:** Louise Freebairn, Jo-an Atkinson, Yang Qin, Christopher J Nolan, Alison L Kent, Paul M Kelly, Luke Penza, Ante Prodan, Anahita Safarishahrbijari, Weicheng Qian, Louise Maple-Brown, Roland Dyck, Allen McLean, Geoff McDonnell, Nathaniel D Osgood, Diabetes in Pregnancy Modelling Consortium, Tracey Baker

**Affiliations:** 1 The Australian Prevention Partnership Centre, Sax Institute, Haymarket, New South Wales, Australia; 2 School of Medicine, The University of Notre Dame Australia, Darlinghurst, New South Wales, Australia; 3 Population Health, ACT Health, Woden, Australian Capital Territory, Australia; 4 Brain and Mind Centre, University of Sydney, Sydney, New South Wales, Australia; 5 Computational Epidemiology and Public Health Informatics Laboratory, University of Saskatchewan, Saskatoon, Saskatchewan, Canada; 6 Endocrinology and Diabetes, ACT Health, Woden, Australian Capital Territory, Australia; 7 Medical School, College of Health and Medicine, Australian National University, Canberra, Australian Capital Territory, Australia; 8 Golisano Children’s Hospital at URMC, University of Rochester, Rochester, New York, USA; 9 School of Computer, Data and Mathematical Sciences, Western Sydney University, Penrith, New South Wales, Australia; 10 Wellbeing and Preventable Chronic Diseases Division, Menzies School of Health Research, Charles Darwin University, Casuarina, Northern Territory, Australia; 11 Endocrinology Department, Royal Darwin Hospital, Casuarina, Northern Territory, Australia; 12 Department of Medicine, University of Saskatchewan College of Medicine, Saskatoon, Saskatchewan, Canada

**Keywords:** gestational diabetes mellitus, modeling, causal modeling, population health

## Abstract

**Introduction:**

Hyperglycemia in pregnancy (HIP, including gestational diabetes and pre-existing type 1 and type 2 diabetes) is increasing, with associated risks to the health of women and their babies. Strategies to manage and prevent this condition are contested. Dynamic simulation models (DSM) can test policy and program scenarios before implementation in the real world. This paper reports the development and use of an advanced DSM exploring the impact of maternal weight status interventions on incidence of HIP.

**Methods:**

A consortium of experts collaboratively developed a hybrid DSM of HIP, comprising system dynamics, agent-based and discrete event model components. The structure and parameterization drew on a range of evidence and data sources. Scenarios comparing population-level and targeted prevention interventions were simulated from 2018 to identify the intervention combination that would deliver the greatest impact.

**Results:**

Population interventions promoting weight loss in early adulthood were found to be effective, reducing the population incidence of HIP by 17.3% by 2030 (baseline (‘business as usual’ scenario)=16.1%, 95% CI 15.8 to 16.4; population intervention=13.3%, 95% CI 13.0 to 13.6), more than targeted prepregnancy (5.2% reduction; incidence=15.3%, 95% CI 15.0 to 15.6) and interpregnancy (4.2% reduction; incidence=15.5%, 95% CI 15.2 to 15.8) interventions. Combining targeted interventions for high-risk groups with population interventions promoting healthy weight was most effective in reducing HIP incidence (28.8% reduction by 2030; incidence=11.5, 95% CI 11.2 to 11.8). Scenarios exploring the effect of childhood weight status on entry to adulthood demonstrated significant impact in the selected outcome measure for glycemic regulation, insulin sensitivity in the short term and HIP in the long term.

**Discussion:**

Population-level weight reduction interventions will be necessary to ‘turn the tide’ on HIP. Weight reduction interventions targeting high-risk individuals, while beneficial for those individuals, did not significantly impact forecasted HIP incidence rates. The importance of maintaining interventions promoting healthy weight in childhood was demonstrated.

**Significance of this study**

**What is already known about this subject?**

- The rising prevalence of hyperglycemia in pregnancy (HIP) is having a significant impact on health service demand and resources, yet the strategies for screening, diagnosing, preventing and managing HIP remain contested.
- Exploration of effective decision support tools is needed to guide evidence-informed policy and programs for this complex problem.

**What are the new findings?**

- The unique tripartite structure of this dynamic simulation model allows representation of the problem and synthesis of evidence at multiple integrated levels of abstraction, including biological, individual-level behavioral and health service dynamics.
- The tested scenarios highlighted the importance of public health interventions to maintain healthy weight status in childhood and support women to achieve healthy weight prior to pregnancy.

**How might these results change the focus of research or clinical practice?**

- Population health interventions will be necessary to stabilize and reduce HIP.
- Interventions targeting high-risk individuals can be beneficial to these individuals however, they delivered small reductions in overall population incidence rates.
- DSMs mature as new evidence becomes available and methods are advanced to facilitate further development.
- A key priority for future research is improved knowledge about the dynamics and heterogeneity in the etiology of glycemic dysregulation and diabetes mellitus development, and the impact of glycemic control during pregnancy on perinatal outcomes.

## Introduction

Hyperglycemia in pregnancy (HIP), inclusive of gestational diabetes and type 1 and type 2 diabetes diagnosed before or during pregnancy, is increasing both in Australia and internationally,^1–4^ challenging the capacity of healthcare services. The increase in HIP is directly associated with the increasing prevalence of risk factors including overweight, obesity, older maternal age and shifts in population demographics and ethnicities.^2 3 5^ With increasing prevalence of risk factors, service providers report that women are more frequently presenting with more complex diabetes and obstetric care needs.^6^ Additionally, diabetes during pregnancy increases the risk for later cardiometabolic disease for the woman^3^ and early onset of overweight, obesity and type 2 diabetes for her children.^2 7^

The available evidence for HIP policy and treatment planning is not definitive,^1^ and current challenges include determining the timing and methods of screening, criteria for diagnosis, targets for treatment, resource allocation, identification and management of pre-existing diabetes during pregnancy, risk stratification, timing and type of prevention activities, and individual differential effects of treatment.^1 8–10^ To address the increasing incidence of HIP, there have been increasing calls for upstream prevention activities to focus on lifestyle risk factors preconception rather than during or interpregnancy.^11–13^ These contested intervention options cross the spectrum from population-based primary prevention approaches to highly specialized clinical management targeted at those at highest risk, which can be implemented independently or in combination and may be phased or implemented simultaneously. Sophisticated analytical tools are required to synthesize diverse evidence types across disciplines and support decision making.

Systems science methods provide decision makers with insights into how multiple causal pathways interact to generate the patterns of disease we see in the real world and how interventions modify those pathways.^14 15^ Dynamic simulation modeling (DSM) is a method to re-create complex systems and human behaviors as a computational mathematical model. This model can answer ‘what if’ questions, via computer simulation, about the likely impacts over time of different policy and intervention options and their combinations.^16 17^ This is important for prevention policy and practice, where decision support tools must steer a course through the complexity of interactions that give rise to real-world public health problems, such as the rapid increase in HIP.^16–18^ They are also useful for conditions with slow and variable development, like diabetes mellitus, that involves interaction between genetic predisposition and environmental factors that impact on the underlying dynamics of physiological factors involved in glucose regulation, such as weight status, insulin sensitivity, insulin secretion and intercurrent pregnancy.^19–21^ These physiological variables interact, most often in non-linear ways, and some are difficult to measure empirically, meaning that conditions like diabetes present significant challenges for traditional experimental methods.^19 22^ Analytical methods like DSM play an important role in improving understanding of the dynamics of disease progression.^19 23 24^ The multiscale, hybrid DSM reported in this paper builds on current understanding of glycemic regulation dynamics related to weight status and pregnancy,^20 21^ leveraging existing peer-reviewed mathematical models of diabetes,^19 23 24^ and explores the dynamics of glycemic regulation, weight status and pregnancy on the development of HIP.^25^

Recent advances in modeling software have increased model transparency, making them more accessible to non-modelers. This has facilitated expert stakeholder participation in the model development process, increasing the opportunities for interdisciplinary learning about complex health problems and building trust in the model outputs.^26–30^ The aim of this study was to develop an HIP decision support tool for policy and program decision makers in the Australian Capital Territory (Box 1), using participatory DSM.^31^ The model development process and discussions of the model outputs enable key stakeholders to explore the likely impacts of both clinical and population-level intervention options for HIP, via simulation, before they are implemented in the real world. The process has been reported elsewhere.^26 30–32^ The aim of this paper is to explore the impact of prevention interventions targeting weight status on HIP incidence and insulin sensitivity. Insulin sensitivity, while not being a commonly used clinical measure, was selected as an outcome measure of glycemic regulation for these simulated scenarios as it reflects metabolic dynamics both during pregnancy and with changing weight status and is potentially responsive to lifestyle interventions.^20 21^ Intervention scenarios were compared with a baseline ‘business as usual’ scenario to explore the impact of timing, subgroup targeting, and adherence to lifestyle changes on the incidence of HIP and insulin sensitivity.

Box 1 **Study context**

- The model explored hyperglycemia in pregnancy (HIP) in the Australian Capital Territory (ACT) and was built in partnership with the ACT Government Health Directorate (ACT Health). Approximately 16% of ACT resident women who gave birth in the ACT in 2016 were diagnosed with HIP (increasing from 6% in 2008).^48^ ACT Health provides government-funded health services for the population of the ACT (approximately 410 000) and is the major health referral center for the Greater Southern Region of New South Wales. The total catchment area population is over 600 000 people. The number of women giving birth in the ACT is over 6000 per year. Approximately 15% of these women are non-ACT residents who access services in the ACT for high-risk pregnancy complications (ie, those requiring tertiary level care). Models of antenatal maternity care provided in the ACT include hospital-based outpatient care, private midwifery care, shared care (ie, integrated with primary healthcare providers) and tertiary level multidisciplinary care. DIP services include a gestational diabetes education program at two sites (hospital and community health center) and a hospital-based, high-risk diabetes in pregnancy multidisciplinary clinic for women with pre-existing type 1 and 2 diabetes and step-up care for women with gestational diabetes requiring insulin or with other complex care needs.

## Methods

### Model development

**Figure 1:**
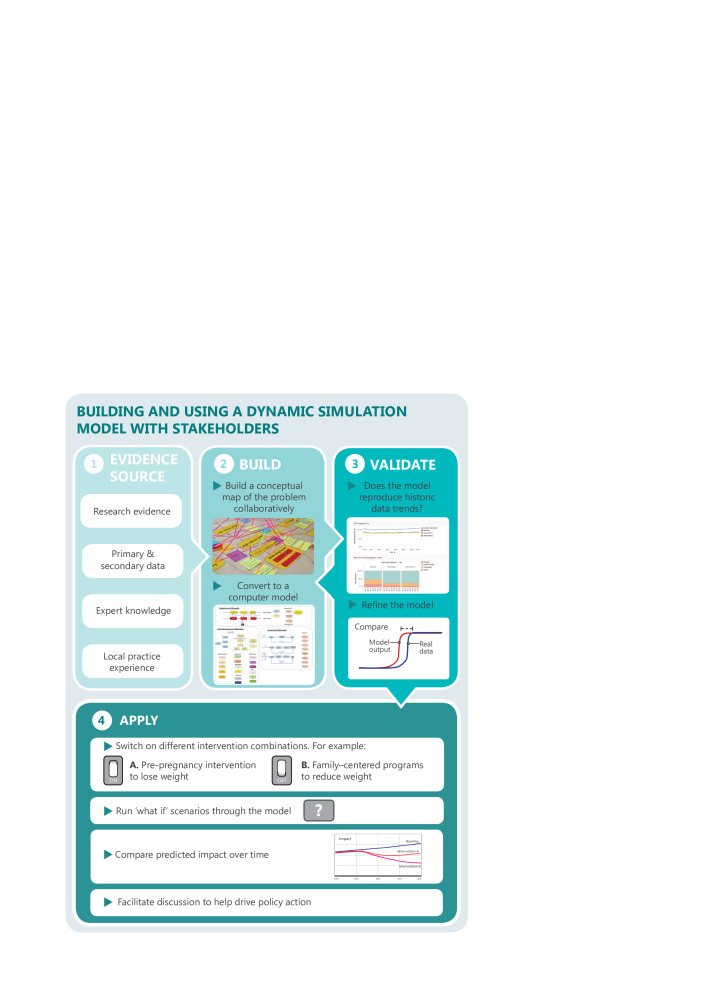
Overview of the participatory model development process.

The model development process drew on best-practice guidelines for computational modeling and included the grounding of assumptions in theory and evidence, sensitivity testing and calibration.^33 34^ The model was built using a participatory approach that engaged a consortium of academics, clinicians, public health policy makers, program planners, modelers and health economists. This approach has been described in detail elsewhere,^26 31 32^ and a diagrammatical overview of the process is depicted in figure 1.

The hybrid model was constructed using AnyLogic simulation software (http://www.anylogic.com/). Detailed information is available in the online supplementary resource.

10.1136/bmjdrc-2019-000975.supp1 Supplementary data

### Model inputs and data sources

The structure and parameterization of the model drew on a range of data sources, including census and population data, systematic reviews, meta-analyses, accepted formulas and conceptual models, survey data, policy/program effectiveness data, economic data and the expert knowledge of the multidisciplinary stakeholders who participated in model development. Local data were prioritized where these were available. Expert opinion was used when other evidence options were exhausted or for triangulation of multiple data sources when parameters were uncertain. The data included statistics relating to demographic characteristics and trends, the incidence of HIP and associated risk factors, and the underlying physiology determining individual glycemic control including beta cell mass and function based on previous mathematical models of diabetes progression.^19 23 24^ Census, population and health system data were sourced from the Australian Bureau of Statistics and ACT Health administrative data collections. Model input parameter values, their sources and the data used for model calibration are provided in the online supplementary resource. The model population is initialized using demographic characteristics, for example, age and country of birth, of the female population of the Australian Capital Territory (ACT) from the 2011 Australian Census.^35^ The model is calibrated to the incidence of HIP in ACT Health maternal and perinatal statistics from 2008 to 2016,^25^ and the model time unit is years.

### Model structure

The tripartite model incorporates system dynamics (SD), agent-based modeling (ABM) and discrete event simulation (DES) components with construction and analysis implemented in AnyLogic V.8.3.3 Professional (http://www.anylogic.com/). SD is a method for understanding the relationships between elements in a system and how the behavior of the system changes^36–39^ using feedback loops (the circular causality in the system), stocks (accumulations/quantities) and flows (rates of change). ABM simulates the actions and interactions of agents (agents are people in this model) to assess their impacts on the system as a whole.^40^ This method is useful for capturing heterogeneity in risk and in impacts of interventions and capturing social network influences. DES methods analyze processes and optimization of resource allocation for service delivery (eg, patient flows through an emergency department).^37^

Underlying the model structure, described here and elsewhere,^25^ are equations and values (parameters) that quantify the relationships defined by the model logic. These are described in detail in the online supplementary resource.

**Figure 2:**
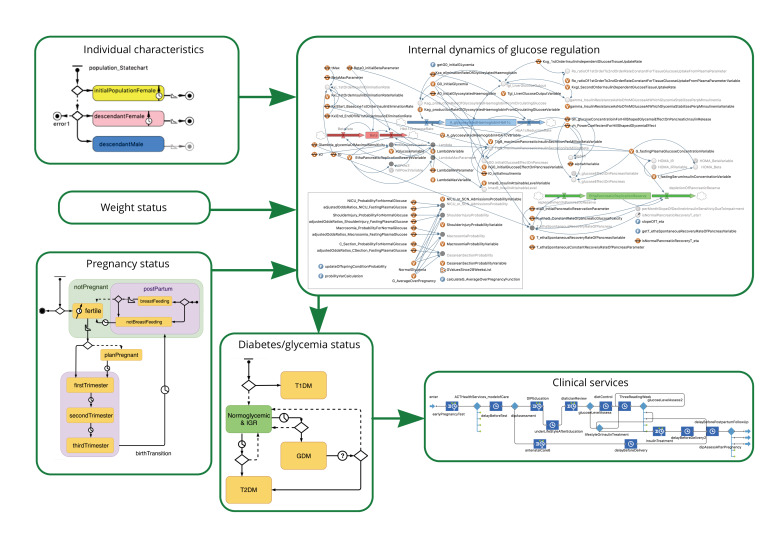
Overview of model components and structure. DIP, Diabetes in Pregnancy; GDM, Gestational Diabetes Mellitus; IGR, Impaired Glucose Regulation; IGT, Impaired Glucose Tolerance; T1DM, type 1 diabetes mellitus; T2DM, type 2 diabetes mellitus.

The overall model structure is shown in figure 2. In summary, the model incorporates characteristics that impact on an individual’s glycemic regulation, including age, individual/family history of diabetes and parity (top left); weight status (center left); and pregnancy status (bottom left). These are represented using agent-based modeling constructs. Glycemic regulation (top right) is represented using SD methods and increases and decreases dynamically. It is impacted by internal physiological factors such as increased metabolic load due to pregnancy, and external behavioral factors such as diet, physical activity and adherence to medication. Diagnosis of HIP (bottom center) leads to clinical service provision (bottom right). Clinical services are represented using DES. Figure 2 is intended to provide a high-level overview of model components to facilitate conceptual understanding of the structure rather than depicting full details. Key components of the model structure are described conceptually in the following paragraphs, and the underlying computational methods are described in the online supplementary resource.

An individual’s risk of developing HIP or progressing on to permanent diabetes relates to the presence or absence (categorical) or level of (continuous) factors known to influence glycemia regulation and is based on previous mathematical models of diabetes progression.^19 23 24^ It is a function of two main groups of factors in the model. First, it is a function of biological regulatory capacity, that is, the changes in insulin sensitivity and insulin production associated with underlying physiology, including in response to the increased metabolic load of pregnancy.^19 21 23 24^ Second, there is a component of external regulation by the individual, that is, their conscious regulation through adherence to blood testing, medication regimens and lifestyle interventions including diet and physical activity. The model mechanism allows for changes in an individual’s adherence to medical and lifestyle interventions over time.

The model also incorporates the impact of beta cell decline associated with exposure to dysglycemia based on modeling carried out by De Gaetano *et al*.^19 23 24^ Exposure to dysglycemia results in a decline of beta cell function over time, and this eventually limits the individual’s regulatory capacity. Reduced beta cell function decreases the effectiveness of lifestyle interventions in glucose regulation, meaning that even if an individual with reduced beta cell function makes significant changes to their diet and activity levels the impact on blood glucose regulation will be restricted.

Pregnancy occurs according to the ACT age and ethnicity specific fertility rates. The model tracks relevant risk factor information for the occurrence of dysglycemia in the current pregnancy, for example, body mass index (BMI), age, history of diabetes, and family history of diabetes. Insulin sensitivity decreases significantly during pregnancy for both normoglycemic and dysglycemic women, based on findings of studies by Catalano *et al*,^20 21 41^ and such changes can impart physiological impact for the mother and the child (eg, on beta cell mass and function) that persists beyond that pregnancy. When a woman gives birth, there is a birth event in which a baby is introduced into the model. The baby inherits information on maternal characteristics, including the mother’s HIP status and history of diabetes, maternal weight status and ethnicity. Outcomes, including birth weight, type of birth, for example, cesarean section, neonatal intensive care admission, and shoulder dystocia, are recorded at birth. Consistent with the focus on HIP, the model includes only female agents. Births for male babies occur in the model; however, these agents are deleted from the population. Model outputs reflect the impact of interventions on women in the population.

High weight status is an important risk factor for declining insulin sensitivity and the development of diabetes. Weight is represented in the model as a continuous variable that changes dynamically with age^42^ and pregnancy.^43^ An individual’s weight status (BMI) impacts on their insulin sensitivity,^19–21^ with increasing weight leading to decreasing insulin sensitivity. This paper reports on weight reduction intervention scenarios tested in the model as described in the next section.

Simplifying assumptions about individual behavior was made to ensure the model is parsimonious, while allowing it to approximate real-world behavior over time. A summary of the key assumptions is presented in the following:

- Age-specific fertility rates were calculated using birth rates from 2013. The model assumes that age-specific fertility rates will remain stable over the period of the simulation.
- The model assumes that 60% of pregnancies were intended, providing opportunities for intervention during pregnancy planning.^11^ The assumption was applied uniformly across age groups.
- Adherence to healthy lifestyle behaviors was assumed to increase after exposure to intervention and then decline over the subsequent 2 years.
- Individuals who were eligible had an equal chance of receiving interventions.

Underlying the model structure and assumptions described are simple mathematical relationships designed to capture the concept they represent. For instance, the decline in intervention adherence was assumed to follow a curve whose coefficients cause adherence to the weight management intervention to increase immediately following an intervention and decline over the subsequent 2-year period.

Health services are captured in the model, with the current service model in the ACT being represented as a DES component. Future planned work for the model will explore the impact of alternative service models on resources and outcomes.

### Scenarios tested in this analysis

The scenarios tested in this analysis focused on the impact of targeted and population-level weight reduction strategies and compared them with a ‘business as usual’ baseline scenario that assumed that existing services and programs would continue and that no additional interventions would be implemented. Many of the risk factors for HIP are not modifiable; however, weight status (overweight and obesity) is an important modifiable risk factor for both HIP and type 2 diabetes mellitus. The scenarios prioritized for this analysis are described in the following sections.

#### Impact of population versus targeted weight management interventions

These scenarios compared the impact of weight management interventions delivered across the population of women aged 20–35 years with targeted interventions delivered to women who were at high risk according to the Australian Diabetes in Pregnancy criteria,^1^ either before or after their pregnancies. The interventions are described in table 1 and are simulated from 2018 in the scenarios.

**Table 1:** Scenario descriptions

Scenario	Description
Population intervention	This intervention targets all women aged 20–35 years through a public health intervention. The goal of the intervention is to support women to maintain or achieve a healthier weight status.
Targeted prepregnancy intervention	This intervention targets women who have one or more risk factor for HIP. It is available to all women who are considering pregnancy (60% of pregnancies^11^). The intervention aims to achieve a healthy weight via adherence to diet and physical activity recommendations.
Targeted interpregnancy	This interpregnancy intervention targets women who have had diabetes in a previous pregnancy. The intervention aims to increase adherence to diet and physical activity recommendations and to achieve a healthy weight before the next pregnancy.
Combined	This scenario combines all the above interventions.

HIP, hyperglycemia in pregnancy.

The effectiveness of each intervention in reducing weight is a model parameter that can be varied. For simplicity, the interventions in these scenario runs were assumed to result in weight reductions that had a truncated normal distribution, with a mean BMI reduction of 1.3 kg/m^2^ (min=0, max=6.4, SD=1.7 kg/m^2^). The distribution was based on an Australian study of mobile phone-based public health intervention aimed at preventing weight gain in young adults^44^ and an Australian study of interpregnancy lifestyle change supported by motivational interviewing.^45^ Weight loss results for individuals who received the interventions were drawn from this distribution. It was assumed that all eligible individuals received the intervention and that the intervention effectiveness degraded over time, with adherence diminishing over a 2-year period.

#### Impact of childhood weight interventions

These interventions explored the impact of childhood weight interventions. As childhood weight dynamics had not yet been fully articulated in the model, these hypothetical scenarios were simulated by modifying the weight distribution of the population on entry to adulthood. Increasing population-wide interventions to reduce childhood overweight and obesity was simulated by shifting the weight distribution of the population to the ‘left’, so that more individuals entered adulthood within the healthy weight range (truncated normal distribution with mean BMI=22). Scaling back population-wide interventions addressing childhood overweight and obesity was also simulated. The scaling back intervention shifted the population weight distribution to the ‘right’, so that more individuals entered adulthood either overweight or obese (truncated normal distribution with mean BMI=30). The interventions were implemented for individuals born from 2018, and the simulations were run for 42 additional years (2060) to allow individuals to age and enter their reproductive years.

### Model outputs and data analysis

For the scenario testing, key outcome indicators against which the impacts of intervention scenarios were compared with the baseline ‘business as usual’ scenario were (1) incidence of HIP (%) and (2) insulin sensitivity (KxgI). Incidence of diabetes in pregnancy was calculated as a percentage based on the proportion of all women giving birth in each year who were diagnosed with HIP. KxgI was used as a mathematical index of insulin sensitivity representing insulin-dependent glucose tissue uptake.^23^

To estimate latent or poorly measured parameters and to support the projection of status quo future incidence of HIP using model outputs, we calibrated the baseline model without additional interventions against the following historical data: the incidence of HIP for subpopulations in ACT from 2008 to 2016 according to the Australian Diabetes in Pregnancy Society (ADIPS) risk profiles^1^; and the prevalence of macrosomia by HIP status in the ACT from 2010 to 2016.^25^

Outputs from the model were summarized using the R statistical package to obtain means, SEs and 95% CIs; summary data were tabulated and graphed in Microsoft Excel. Given that runs of the model were computationally expensive, 36 runs were deemed sufficient to account for stochasticity and provide stable predictions of scenario performance and of the variance in performance. The comparison of simulation results between baseline and intervention scenarios was expressed as a per cent difference in reported outcomes, with 95% CIs provided to describe the variation between simulation runs and to test statistical significance.

## Results

Results for scenario testing for interventions with simulated implementation from 2018 are presented in the following sections.

### Scenario testing results

#### Impact of population versus targeted weight management interventions

**Figure 3:**
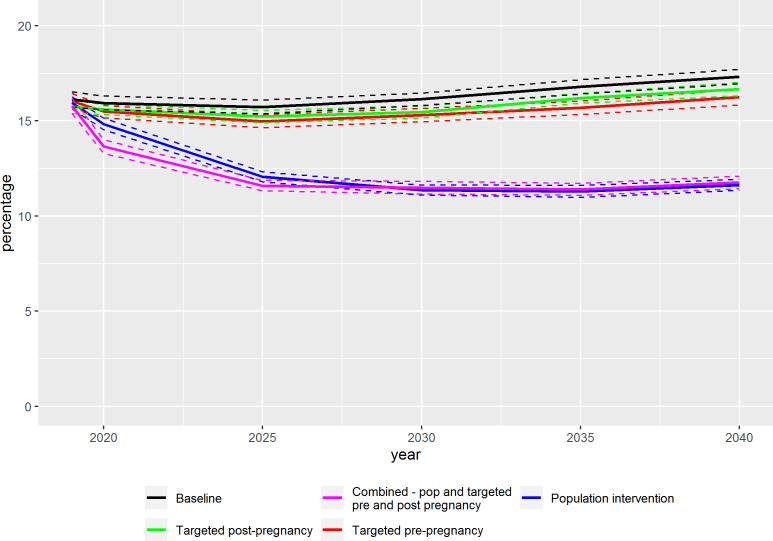
Comparative impact of scenarios on HIP incidence simulated from 2018 to 2040. Dotted lines indicate 95% CI for estimated incidence. HIP, hyperglycemia in pregnancy.

The incidence of HIP for the scenario simulations is presented as a percentage, based on the proportion of all women giving birth in each year who were diagnosed with HIP, in table 2. The incidence of HIP in the baseline scenario was 15.9% (95% CI 15.5 to 16.3) in 2020, 16.1% (95% CI 15.8 to 16.4) in 2030 and 17.3% (95% CI 16.9 to 17.7) in 2040. The population weight loss intervention in early adulthood resulted in a 3.0% reduction in HIP incidence by 2020 to 15.5% (95% CI 15.1 to 15.9), which falls within the margin of error of the baseline; however, by 2030 there was a 17.6% reduction against the baseline with an HIP incidence of 13.3% (95% CI 13.0 to 13.6) (figure 3). In comparison, the impact of targeted prepregnancy and interpregnancy interventions on population-level HIP incidence ranged from a non-significant reduction of just over 2% in 2020, to a small but statistically significant reduction of 4%–5% in 2030 and 4%–6% in 2040, respectively. Incidence rates with CIs for these scenarios are presented in table 2. Combining targeted interventions for high-risk groups with population weight loss interventions was the most effective scenario for reducing HIP incidence, with a reduction of 14.4% by 2020 to 13.6% (95% CI 13.2 to 14.0), 2 years after the simulated interventions were implemented, 28.8% by 2030 (HIP incidence=11.5%, 95% CI 11.2 to 11.8) and 32.1% by 2040 (HIP incidence=11.8%, 95% CI 11.5 to 12.1).

**Table 2:** Summary HIP incidence statistics for baseline and scenarios simulated from 2018 to 2040

	2020			2030			2040		
	%	95% CI (±)	% reduction from baseline	%	95% CI (±)	% reduction from baseline	%	95% CI (±)	% reduction from baseline
Baseline	15.9	0.4	–	16.1	0.3	–	17.3	0.4	–
1. Population intervention	15.5	0.4	−3.0	13.3*	0.3	−17.6	13.8*	0.3	−20.5
2. Targeted prepregnancy	15.5	0.3	−2.8	15.3*	0.3	−5.2	16.2*	0.4	−6.2
3. Targeted interpregnancy reduction	15.6	0.3	−2.1	15.5*	0.3	−4.2	16.7*	0.4	−3.8
4. Combined population and targeted prepregnancy and interpregnancy	13.6	0.4	−14.4	11.5*	0.3	−28.8	11.8*	0.3	−32.1

*Significantly different from baseline at p<0.05.

HIP, hyperglycemia in pregnancy.

#### Impact of childhood weight status on entry to adulthood

The interventions were implemented for female agents born from 2018 and were simulated to 2060 to allow time for individuals to age into adulthood and their reproductive years. Two scenarios were simulated: scenario A—shifting the BMI distribution of the population to healthy weight with all individuals entering adulthood at a healthy weight; and scenario B—shifting the weight distribution for the population further toward overweight and obesity as they entered adulthood. Minimal impact of the interventions was observed on HIP incidence until 2060, when scenario A resulted in a 21.2% decrease in the percentage of women diagnosed with HIP from baseline (table 3) (2060 baseline HIP incidence=17.0%, 95% CI 16.7 to 17.3; scenario A HIP incidence=13.4%, 95% CI 13.1 to 13.7; scenario B HIP incidence=17.6%, 95% CI 17.3 to 17.9).

**Table 3:** Summary HIP (percentage) incidence and population insulin sensitivity (KxgI) statistics for baseline and scaling up and scaling back scenarios simulated from 2018 to 2060

HIP incidence (%)	2020			2030			2040			2050			2060		
	%	95% CI (±)	% change from baseline	%	95% CI (±)	% change from baseline	%	95% CI (±)	% change from baseline	%	95% CI (±)	% change from baseline	%	95% CI (±)	% change from baseline
Baseline	15.6	0.28		15.8	0.31		16.9	0.31		17.2	0.3		17.0	0.3	
Scenario A: all normal weight	15.9	0.26	1.8	15.9	0.31	0.8	17.1	0.31	1.4	17.0	0.3	−0.9	13.4*	0.3	−21.2
Scenario B: more overweight or obese	15.7	0.27	0.4	15.7	0.30	−0.6	17.1	0.27	1.3	17.3	0.3	0.4	17.6	0.3	3.8

Insulin sensitivity (KxgI)	2020			2030			2040			2050			2060		
	Mean	95% CI (±)	% change from baseline	Mean	95% CI (±)	% change from baseline	Mean	95% CI (±)	% change from baseline	Mean	95% CI (±)	% change from baseline	Mean	95% CI (±)	% change from baseline
Baseline	50.3	0.02		48.4	0.02		46.4	0.03		45.7	0.02		45.6	0.02	
Scenario A: all normal weight	50.9	0.02	1.2	52.5	0.02	8.5	54.2	0.03	16.9	60.0	0.03	31.2	67.1	0.04	47.3
Scenario B: more overweight or obese	49.7	0.02	−1.2	44.6	0.02	−7.8	39.3	0.02	−15.4	34.7	0.02	−24.1	31.5	0.02	−30.9

KxgI: an index of insulin sensitivity representing insulin-dependent glucose tissue reuptake.

*Significantly different from baseline at p<0.05.

HIP, hyperglycemia in pregnancy.

Changes in insulin sensitivity (KxgI) were observed earlier in the simulation, from 2030, for the simulated BMI interventions (table 3). Scenario A resulted in increased insulin sensitivity, as measured by KxgI, for the population by 8.5% from the baseline simulation by 2030 (baseline KxgI=48.4, 95% CI 48.2 to 48.6; scenario A KxgI=52.5, 95% CI 52.3 to 52.7), increasing to 47.3% by 2060 (baseline KxgI=45.6, 95% CI 45.4 to 45.8; scenario A KxgI=67.1, 95% CI 66.7 to 67.5). Scenario B resulted in a decrease in insulin sensitivity for the population of 31% from baseline by 2060 (scenario B KxgI=31.5, 95% CI 31.3 to 31.7).

## Discussion

The simulations reported here prioritized scenario testing of several lifestyle prevention interventions promoting healthy weight status. Population-level interventions promoting weight loss in early adulthood were found to be more effective than targeted prepregnancy and interpregnancy interventions in reducing the population incidence of HIP. Combining targeted interventions for high-risk groups with population health promotion support was shown to be the most effective scenario for reducing HIP incidence, especially in the longer term. Scaling up childhood healthy weight interventions, resulting in all female children entering adulthood at a healthy weight, achieved a significant improvement in insulin sensitivity in the short term and decreased HIP in the long term. Scenarios testing the impact of scaling back childhood healthy weight interventions, that is, having more children entering adulthood overweight or obese, resulted in declines in insulin sensitivity across the population and therefore increasing risk of early development of diabetes mellitus.

The study presented in this paper is unique in that DSM was used to explore the latent factors and metabolic dynamics underlying the development of HIP and compare the likely impact of population-level interventions with interventions targeting high-risk individuals. This simulation study builds on previous research assessing the effectiveness of targeted lifestyle prevention programs to prevent HIP incidence.^11 13 46^ Given the substantial time needed to achieve weight reduction, it has been argued that early intervention at a population level will be necessary to reduce obesity-related outcomes in pregnancy,^11^ and this was supported by the modeling. The scenarios presented in this paper demonstrated that population-level interventions will be needed to make an impact on HIP incidence across the population. Targeted interventions, both prepregnancy and interpregnancy, did not substantially impact on population HIP incidence.

Over half of pregnancies are planned,^11^ and this was reflected in the model, with only individuals who were planning to become pregnant being eligible to receive the targeted preconception intervention. Therefore, the small proportion of the total population receiving the intervention and individual variations in adherence, included in the model to reflect reality, impacted on intervention effectiveness. The targeted interventions resulted in only a modest impact on population incidence rates for HIP. This result should not devalue the role of targeted interventions, as these are important and beneficial for individuals and their offspring.^12^ However, the results emphasize the need for population interventions to support healthy lifestyle behaviors for all individuals, whether they actively plan their pregnancy or not.^11^

A recent review of research into antenatal lifestyle programs for high-risk women found that they did not successfully prevent HIP.^13^ Further examination of the individual and intervention characteristics that facilitated adoption and adherence to interventions has been identified as a priority.^13^ The HIP model presented here incorporated representations of the non-linear dynamics and feedback loops that impact intervention effectiveness, for example, the impact of age and pregnancy-related weight changes across the life course and the impact of individual adherence to diet and physical activity recommendations on both HIP incidence and insulin sensitivity. The reduction in HIP incidence was only achieved when individuals remained adherent to the lifestyle changes associated with the intervention.

Scenario testing provides an important tool for exploring hypothetical policy options, including ‘do nothing’ alternatives that forecast the impact of ceasing current interventions.^15 33 47^ In these simulated scenarios, the HIP model hypothetically tested the impact of scaling back interventions promoting healthy weight for children in school settings. This scenario forecasted the impact of more children entering adulthood at a higher weight status on insulin sensitivity, placing them at risk of early development of diabetes mellitus. These results signify the potential importance of the current global focus and efforts to reduce childhood overweight and obesity.

Diverse local perspectives and interests can provide decision makers with conflicting advice regarding the best course of action.^17^ Data limitations, insufficient local analytical capacity and inadequate tools to support longer term planning in the context of changing local needs contribute to the persistence of a trial and error approach to program planning that may delay or prevent the realization of significant impacts on important public health issues like HIP.^16 47^ The DSM approach described in the present study is one way to address these challenges and can also contribute to prioritizing data gaps for future research and data collection, and infrastructure to better support interventions to prevent and manage HIP. The participatory approach facilitated opportunities for interdisciplinary dialogue and combining diverse perspectives in the consideration of policy options. The developed partnerships and relationships were critical to the model development and to its likely subsequent use to inform health service and policy decisions.

Future applications of the model include further exploration of the intergenerational impacts resulting from exposure to HIP; effect of glycemic dysregulation on pregnancy outcomes; impact of lifestyle (diet and physical) interventions during pregnancy on glycemic control and pregnancy outcomes; factors that influence childhood weight gain, for example, breast feeding and other aspects of diet, school-based health promotion interventions, physical activity and so on; impact of model of care alternatives; and impact of prevention interventions on health service utilization. Health economic considerations will also be added to future iterations of the model.

### Limitations

There are limitations to consider when interpreting the findings of this paper. There is potential measurement bias in the range of secondary data used to parameterize the model. Where possible, routinely collected local health service information was obtained to estimate population-based estimates of HIP, birth outcomes, weight status, and fertility rates. There were also some parameters relating to the heterogeneity of etiology of HIP and the dynamics of glycemic regulation where data were not available, and these are identified as priorities for future research. The model acknowledges these potential sources of measurement bias, and commonly used strategies were employed to address them, including the triangulation of multiple data sources, calibration to refine parameter estimates and the engagement of stakeholders with detailed knowledge of the limitations and likely direction and size of potential measurement biases in key data sources. In addition, sensitivity analysis was undertaken to estimate the impact of uncertainty on primary outcome indicators and guide priorities for new data collection and quality improvement of existing data collection.

## Conclusion

Population health interventions will be necessary to ‘turn the tide’ on HIP. Interventions targeting high-risk individuals, while beneficial for those individuals, delivered small reductions in HIP incidence rates. The importance of maintaining interventions promoting healthy weight in childhood was demonstrated. Scenarios simulating the impact of scaling back these interventions showed that insulin sensitivity decreased significantly, increasing the risk for early development of diabetes mellitus. DSMs are learning support tools that can mature over time as new evidence becomes available and methods are advanced to facilitate further development. This decision support tool for HIP was developed as a working model and is being published for transparency and to invite input. A key priority for future research is improved knowledge about the dynamics and heterogeneity in the etiology of glycemic dysregulation and diabetes mellitus development, and the impact of glycemic control during pregnancy on perinatal outcomes.
